# A Pilot-Scale Supercritical Carbon Dioxide Extraction to Valorize Colombian Mango Seed Kernel

**DOI:** 10.3390/molecules26082279

**Published:** 2021-04-14

**Authors:** Leidy J. Cerón-Martínez, Andrés M. Hurtado-Benavides, Alfredo Ayala-Aponte, Liliana Serna-Cock, Diego F. Tirado

**Affiliations:** 1 Grupo de Investigación en Tecnologías Emergentes en Agroindustria (TEA), Agroindustrial Engineering Faculty, Universidad de Nariño, 1175 Pasto, Colombia; leidyceron07@gmail.com (L.J.C.-M.); ahurtado@udenar.edu.co (A.M.H.-B.); 2 School of Food Engineering, Universidad del Valle, 760031 Cali, Colombia; alfredo.ayala@correounivalle.edu.co; 3 School of Engineering and Administration, Universidad Nacional de Colombia, 763533 Palmira, Colombia; lserna@unal.edu.co; 4 Grupo de Investigación en Innovación y Desarrollo Agropecuario y Agroindustrial (IDAA), Universidad de Cartagena, Campus Piedra de Bolívar, 130015 Cartagena de Indias, Colombia

**Keywords:** *Mangifera indica* L., circular economy, valorization strategy, fruit by-products, response surface methodology, essential fatty acids

## Abstract

Colombian mango production, which exceeded 261,000 t in 2020, generates about 40% of the whole fruit as solid waste, of which more than 50% are seed kernels (over 52,000 t solid by-product); though none is currently used for commercial purposes. This study reports the results of the supercritical carbon dioxide (scCO_2_) extraction of an oil rich in essential fatty acids (EFAs) from revalorized mango seed kernels and the optimization of the process by the Response Surface Methodology (RSM). In pilot-scale scCO_2_ experiments, pressure (23–37 MPa) and temperature (52–73 °C) were varied, using 4.5 kg of CO_2_. The highest experimental oil extraction yield was 83 g/kg (37 MPa and 63 °C); while RSM predicted that 84 g/kg would be extracted at 35 MPa and 65 °C. Moreover, by fine-tuning pressure and temperature it was possible to obtain an EFA-rich lipid fraction in linoleic (37 g/kg) and α-linolenic (4 g/kg) acids, along with a high oleic acid content (155 g/kg), by using a relatively low extraction pressure (23 MPa), which makes the process a promising approach for the extraction of oil from mango waste on an industrial scale, based on a circular economy model.

## 1. Introduction

The concept of sustainability relies on the circular economy model, which focuses on resource efficiency, waste reduction, recycling, and valorization [1,2]. Food waste is a key element of the circular economy concept, and the valorization of these wastes constitutes a promising alternative to turn waste into valuable bio-based products [2,3]. In this regard, the extraction of high-value functional compounds offers one of the many valorization strategies for a sustainable and efficient waste management approach, with possible benefits for the food, pharmaceutical, and cosmetic industries [2].

Mango (*Mangifera indica* L.) is one of the most important and popular tropical fruits worldwide [4,5], ranking second in terms of production and cultivated area, after bananas. Mango had a global production volume of over 55 million t in 2020 and is projected to reach 65 million t by 2028, increasing at an annual rate of 2.1% over this decade [6]. Approximately 99% of mango production takes place in developing countries, with Latin America contributing 26% of production, led by Peru, Brazil, and Colombia [6,7].

The mango processing industry produces a large amount of solid waste as peels, pomace, and seed kernels, which altogether account for 40% to 60% of the whole fresh fruit, of which up to half may be seed kernels [8]. Colombian mango production exceeded 261,000 t in 2020 [9], which indicates that the country had a production capacity of more than 156,000 t of solid waste last year. Up to 78,000 t of those residues were seed kernels, none currently used for commercial purposes, even though the mango seed kernel contains several families of health-promoting compounds such as fatty acids [10] and other substances (triacylglycerols, gallotannins, xanthones, flavonoids, phenolic acids, among others) with related health benefits and interesting techno-functional properties for applications in the food [8,11] and pharmaceutical industries [12]. Therefore, through the use of revalorization strategies, based on the circular economy model, these by-products could be used as a source of important bioactive compounds with high added value.

Likewise, the use of supercritical fluids as alternative solvents for oil extraction has been attracting widespread interest due to their particular properties (e.g., liquid-like solvent power, negligible surface tension, and gas-like transport properties) and changes in environmental regulations, which foster the utilization of green solvents [13]. In this field, carbon dioxide (CO_2_) has been specially employed [14,15] since it is essentially non-toxic, non-flammable, inexpensive at the industrial level, can be recycled, has easily accessible supercritical conditions, and is dissipated from extracts at atmospheric pressure, avoiding the necessity of further expensive and harmful refining treatments [16]. Moreover, extractions with supercritical CO_2_ (scCO_2_) have been successfully recognized in the obtention of valuable extracts from agro-industrial by-products [17].

On the other hand, fatty acids are divided into two main groups according to their structural characteristics: saturated (SFAs) and unsaturated fatty acids (UFAs). The latter, depending on their degree of unsaturation, can be classified as mono (MUFAs) and polyunsaturated fatty acids (PUFAs). Depending on the position of the double bond, counting from the extreme carbon to the carboxylic functional group, MUFAs and PUFAs can be classified into three main series: omega-9 (ω-9, first double bond in carbon 9), omega-6 (ω-6, first double bond in carbon 6), and omega-3 fatty acids (ω-3, first double bond in carbon 3) [18]. Essential fatty acids (EFAs) are those PUFAs that must be provided by foods because these cannot be synthesized in the body yet are necessary for health. There are two families of EFAs: ω-6 and ω-3. Linoleic (C_18:2_ *cis*-9,12) and α-linolenic acids (C_18:3_ *cis*-9,12,15) are examples of ω-6 and ω-3, respectively [19]. Currently, an awareness of the role of these EFAs in human health and disease prevention has been unremittingly increasing among people, and many studies have positively correlated these compounds with infant development, cancer prevention, optimal brain and vision functioning, and the reduction of arthritis, hypertension, cardiovascular morbidity and mortality, diabetes mellitus, and neurological/neuropsychiatric disorders [20]. Therefore, the search for sustainable resources for these compounds is paramount. Moreover, the global market for natural fatty acids was US $13.5 billion in 2018 and is expected to reach US $ 17.5 billion by 2023. A growing demand for naturally derived products in the food, beverage, and dietary supplement industries is expected to be a significant driving force in the future global market [21].

Some studies have employed the special properties of scCO_2_ to achieve the valorization of mango seed kernels [22,23,24]. As an example, Awolu et al. [22] reported an oil extraction yield between 25 g extract/kg biomass and 36 g extract/kg biomass by scCO_2_ extraction from the agri-food industrial by-product. Other studies also reported low oil extraction yields at the same conditions used in this work [23,24]; and to the best of our knowledge, no study has focused on the pilot-scale scCO_2_ production of lipid fractions rich in EFAs from mango seed kernels.

The search for natural sources of bioactive compounds with nutritional interest has received widespread attention, so research in this field has explored the valorization of residues from conventional processes [8,23,25]. These target compounds have usually been extracted using organic solvents; however, this technique is not selective and has become less attractive as it generates products with traces of the residual solvent [26]. The aforementioned, along with the growing awareness of the environmental and health problems arising from the use of organic solvents has led to the implementation of tougher regulations; hence, the use of scCO_2_ extractions has become a commercial and environmentally friendly alternative [14].

In this work, first, the main operating conditions were optimized to achieve a high oil extraction yield from mango seed kernels in a pilot-scale scCO_2_ plant. Afterward, the focus was placed on a selective extraction of a lipid fraction rich in EFAs.

## 2. Results and Discussions

The current work proposed a valorization strategy of mango seed kernels. For this, scCO_2_ experiments at a pilot-scale were performed in a wide range of pressures (from 23 up to 37 MPa) and temperatures (from 52 to 73 °C) by using a solvent ratio of 22.5 kg CO_2_/kg biomass. Pressure and temperature were analyzed as the main operating conditions of the procedure and optimized by Response Surface Methodology (RSM) to reach a high oil extraction yield with a fatty acid profile rich in EFAs (mainly linoleic and α-linolenic acids). Table 1 shows the rotatable central composite design (RCCD) conditions and scCO_2_ extraction yields from the studied biomass.

### 2.1. Total Extraction Yield Analysis

As can be seen in Table 1, the extraction yield reached at the most adequate experimental conditions with supercritical extraction (37 MPa and 63 °C) was 83 g extract/kg mango seed kernels. Supercritical extracts were semi-solid at ambient temperature and light white in color, physically cleaner than the Soxhlet extracts. Meanwhile, the conventional Soxhlet extraction with hexane had a total extraction yield of 90 g extract/kg mango seed kernels. This slightly higher oil extraction yield with the organic solvent was probably achieved due to the lower selectivity of the Soxhlet procedure since the extract obtained with hexane contained neutral and polar lipids and pigments like chlorophyll [25]. This explains the differences in appearance between an extract obtained by one method (scCO_2_) and the other (Soxhlet). Besides, it should be noted that the plant material was not completely exhausted during the scCO_2_ extraction, which also explains the oil extraction yield differences.

### 2.2. Influence of Solid Conditions on Extraction Yield

The highest extraction yield value found in this work (83 g extract/kg mango seed kernels) was within the range of oil extraction values reported by other authors for scCO_2_ extractions from mango seed kernels [23,24]. Jahurul et al. [23] reported an extract yield of 110 g extract/kg mango seed kernels using 50 MPa and 60 °C. Meanwhile, in another study [24], the same authors reported that the highest yield was roughly 130 g extract/kg biomass at 42.2 MPa and 72 °C. However, those other studies involved higher pressures (and therefore more energy expenditure) to achieve an extraction yield close to the one found in the current work, which used a lower pressure (37 MPa). The aforementioned could be due to the improved solid conditions of the biomass subjected to supercritical extraction in this study [25,27], since an optimal particle size and moisture content were pursued in prior experiments, which were then applied during the high-pressure procedure.

During the scCO_2_ extraction from a solid substrate the mass transfer depends heavily on the transport rate in the solid phase and the length of the transport path through the solid [27,28]. Solid particles with a diameter greater than 0.80 mm hinder the penetration of the supercritical solvent and the solubilization of the solute, while particles smaller than 0.4 mm could represent a risk of formation of preferential channels and blockages during extraction [25,28]. Therefore, seed kernels with a particle size of 0.6 mm were subjected to supercritical extractions during the current work. In contrast, particles smaller than 0.2 mm were used in the studies of Jahurul et al. [23,24].

On the other hand, the excess of water can create a barrier to mass transfer when the raw material is subjected to scCO_2_ extraction [27,28]. Therefore, the initial water content of the biomass was also optimized. In our case, preliminary studies showed that the fastest extraction was achieved when the sample had a water mass fraction of 6.8%, which was the equilibrium moisture with the environmental humidity in Pasto. However, to improve the extraction rate it was necessary to dry the raw material, which should be taken into account for the economic viability of the whole process.

### 2.3. Effect of Process Parameters on Oil Extraction Yield

The statistical significance of scCO_2_ pressure and temperature, and their interactions, on mango seed kernel oil extraction yield, was assessed by analysis of variance (ANOVA). Statistically significant effects (*p* ≤ 0.05) on the extraction yield were found for the pressure levels employed, the pressure-temperature interaction, and squares of both pressure and temperature. According to ANOVA, the temperature did not show a significant effect under the levels evaluated.

**Figure 1:**
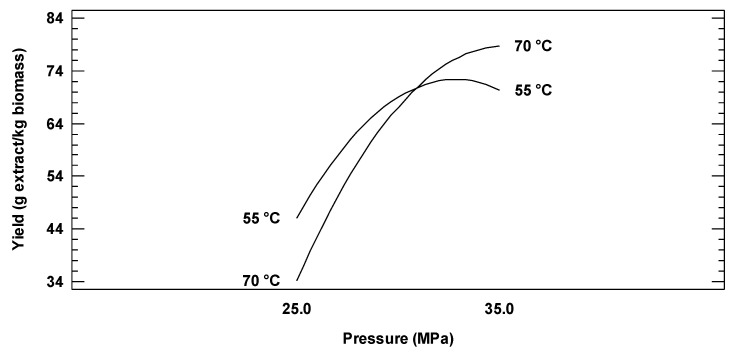
Effect of pressure and temperature on oil extraction yield from mango seed kernels.

As expected, extraction yields significantly increased with pressure (see Figure 1). As shown in Figure 1, by increasing pressure from 20 to 35 MPa, the oil extraction yield increased significantly at the temperature levels evaluated, which was related to the increase in solvent density and solvent capacity of scCO_2_ [27]. Also, by increasing pressure, the solvent penetration into seed pores was enhanced, thus achieving an efficient contact between scCO_2_ and the substrate [28].

As seen in Figure 1, the temperature-pressure interaction played a special role during scCO_2_ extractions from mango seed kernels in this study. The isotherms shown in Figure 1 intersected at a point called the crossover point (slightly over 30 MPa), which represents the pressure at which the increase of the operating temperature benefited the extraction [28]. As a result, the pressure-temperature interaction during the scCO_2_ extraction of this study had a statistically significant effect (*p* ≤ 0.05). For vegetable oils the crossover pressure is about 30 MPa [29], so the crossover point found for mango seed kernel oil in this study was within the values reported in the literature.

During a scCO_2_ extraction, working at constant pressure, the change in temperature affects the density of CO_2_, the vapor pressure of the solutes involved in the extraction procedure, and their desorption from the biomass [25]. At higher temperatures, the solute becomes more volatile; however, the scCO_2_ density decreases [28,29]. In Figure 1, at a relatively low pressure (25 MPa), and below the crossover point, a decrease of density and solvent power with increasing temperature prevailed, while an increase in vapor pressure predominated at a fairly high pressure (35 MPa). This phenomenon is an example of how the optimal temperature to increase oil yield should be well established. It is not possible to anticipate the temperature impact, as it affects many different aspects of the thermodynamics and mass transfer during the high-pressure procedure [25,28].

Even though the extraction yield obtained by the conventional solvent extraction did not differ significantly from the scCO_2_ extraction, it should be noted that quite a high extraction pressure (37 MPa) was required during the extraction to achieve an extract yield of 83 g extract/kg biomass (see Table 1). Considering that the pilot extraction of this study constitutes preliminary data for a future profitable industrial extraction from mango seed kernels, this matter should be addressed.

### 2.4. Oil Extraction Yield Optimization

**Figure 2:**
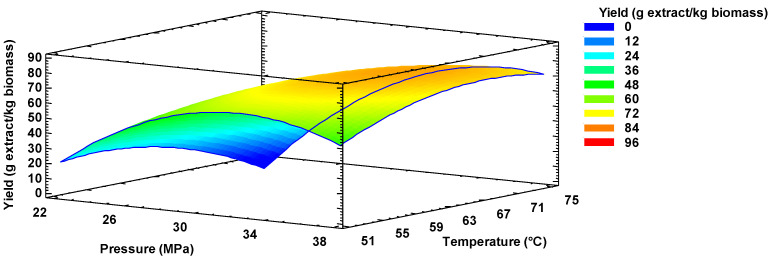
Optimization of the mango seed kernel oil extraction yield with scCO_2_ by Response Surface Methodology (RSM).

RSM predicted a maximum oil extraction yield from mango seed kernels of 84 g extract/kg biomass, operating at 35 MPa and 65 °C (see Figure 2). It is worth noting that, according to the prediction, with a reduction in operating pressure and a minor reduction in temperature, an oil yield slightly higher than that already obtained experimentally could be achieved. The possible explanation for the above phenomenon is that excessive pressures hinder mass transfer and thus extraction of the extract [28].

The optimization provided the second-order mathematical model for the response surface of Figure 2, as expressed in Equation (1):

(1) $$Y=-51.9706+1.96210X_{1}+0.782824X_{2}+0.0121X_{1}X_{2}-0.0393747X_{1}^{2}-0.0091667X_{2}^{2}$$

where Y is the experimental extraction yield (g/kg); and X_1_ and X_2_ are the operating pressure and temperature, respectively. This model fitted the factors studied at their actual physical levels and explained 96% of the variability of the mango seed kernel oil yield data. The optimal statistical extraction conditions were within the range of the experimentally evaluated levels. Finally, the lack-of-fit test was not significant (*p* > 0.05); and therefore, the model was adequate to describe the observed data.

### 2.5. Fatty Acid Profile

Table 2 contains the fatty acid profile of the lipid extracts obtained by supercritical extraction and by conventional extraction with hexane. The fatty acid profile of the lipid fraction revealed the presence of palmitic, stearic, oleic, linoleic, and α-linolenic acids. The content of each fatty acid in the obtained extract depended on the method and the supercritical extraction conditions.

The mango seed kernel oil obtained with scCO_2_ was, on average, made up of a higher proportion of UFAs (about 58%) than SFAs (about 42%); with a variation in the ratio of unsaturated/saturated fatty acids from 1.09 to 2.45, depending on the extraction conditions and extraction method. Table 2 shows that, regardless of the extraction method or condition, oleic acid was the major constituent of the extracts. Oleic acid content was much higher in supercritical extracts than in those obtained with hexane, representing up to almost 50% of the lipid fraction.

Oleic acid is an ω-9 fatty acid. Unlike ω-3 and ω-6, ω-9 fatty acids are not essential since humans can introduce an unsaturation to an SFA in that position. Therefore, oleic acid, to which beneficial nutritional properties are attributed, does not need to be present in our diet [18]. However, it is beneficial to obtain oleic acid from food because similar to other ω-9 fatty acids, oleic acid can help to reduce the risk of heart diseases by raising levels of high-density lipoprotein and lowering low-density lipoprotein [30]. It is not the same with ω-3 and ω-6 fatty acids since our organism cannot introduce unsaturations in these positions. In this way, fatty acids such as linoleic and α-linolenic acids are essential; so our diet requires them in well-determined proportions as their lack or imbalance in intake produces serious metabolic alterations [31].

As seen in Table 2, extracts obtained in this study contained a considerable fraction of both linoleic and α-linolenic acids, which provides additional value in terms of nutritional quality to the lipid fractions obtained in this proposal for the revalorization of mango seed kernels. Table 2 shows that the profile of these acids in the supercritical extracts exceeded that of the extracts obtained with hexane, and the supercritical extracts obtained at 23 MPa and 63 °C were particularly noteworthy. At first, the good fatty acid profile of the supercritical extracts obtained in preliminary tests was surprising, which was another reason for the decision to explore different extraction conditions with an RCCD experimental design; and thus, to find the best-operating conditions to obtain an extract rich in these two EFAs. scCO_2_ at 23 MPa and 63 °C yielded the best results. More importantly, no excessive pressures were needed to achieve this selective extraction.

Although previous studies claimed the presence of linoleic and α-linolenic acids in supercritical extracts from mango seed kernels [23,24,32]; those studies reported lower values than these found in this study in terms of both compounds. Indeed, studies such as that of Ballesteros-Vivas et al. [8] did not report the presence of α-linolenic acid in their extracts. The above could be due to the optimization of the operating and the solid conditions of this study [25,27]; but it could also be due to the mango variety involved in each study [23].

Finally, palmitic and stearic acids accounted for about 15% and 22% of the fatty acid fraction of the supercritical extracts, respectively (see Table 2). The presence of these compounds as major fatty acids in mango seed kernel extracts has been previously reported [23,24,32]. In terms of future industrial applications of the obtained extracts, their presence offers stability, yielding a cocoa butter analogy fat and a product with characteristics of vegetable butter, suitable for use in the confectionery industry [23,24,33].

### 2.6. Extraction Optimization of Targeted Fatty Acids

The extraction pressure, along with the pressure-temperature interaction, had a statistically significant effect (*p* ≤ 0.05) on the extraction of oleic, linoleic, and α-linolenic acids. Temperature, on the other hand, had a statistically significant effect (*p* ≤ 0.05) only on the extraction of oleic acid.

For all the analyzed fatty acids, the pressure exerted a negative effect on the extraction since the increase in the scCO_2_ extraction pressure led to a decrease in the diffusion coefficient of the extracted fatty acids. According to López-Padilla et al. [34], when solvent density increases due to high pressure, diffusion becomes more difficult due to a higher number of molecular collisions. According to those authors [34], intermolecular interactions also increase, since the average intermolecular distance decreases as the density increases, which interferes with the extraction of fatty acids.

As already mentioned, the pressure-temperature interaction was also significant (*p* ≤ 0.05) during the extraction of oleic, linoleic, and α-linolenic fatty acids. It was established that the effect of temperature at low pressures (25 MPa) was positive, while at high pressure (35 MPa), an increase in temperature led to a decrease in the extraction yield of fatty acids. This was since the diffusion coefficient and solubility of fatty acids increased with increasing temperature at a constant pressure. This effect was less significant at higher pressures [34].

**Figure 3:**
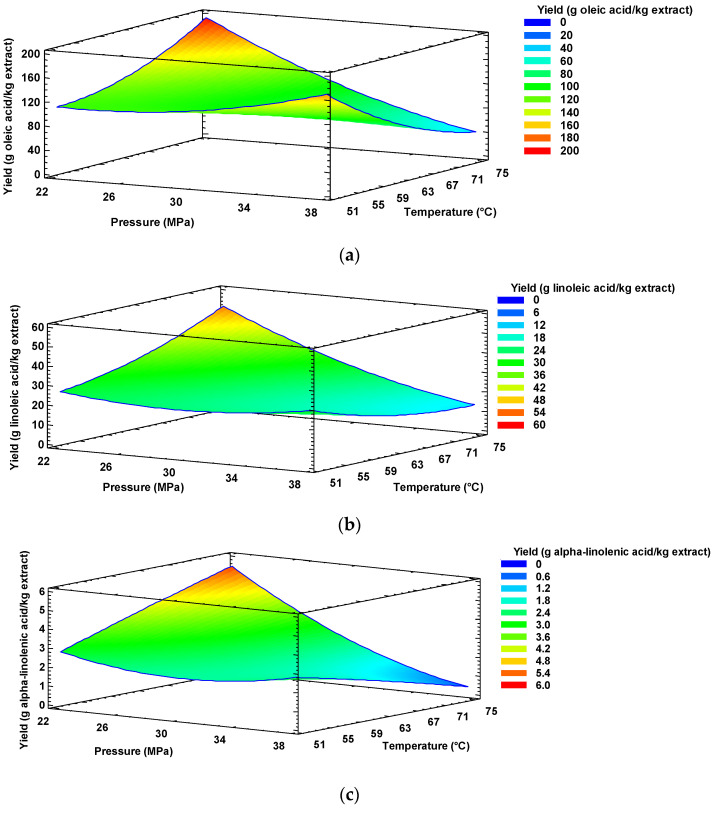
Effect of temperature and pressure on the (**a**) oleic, (**b**) linoleic, and (**c**) α-linolenic acid extraction yield from mango seed kernels.

Response surfaces in Figure 3 show the influence of extraction pressure and temperature on the oleic, linoleic, and α-linolenic acid contents in supercritical extracts. According to Figure 3, it could be established that the highest concentration of the three fatty acids could be obtained in lipid fractions extracted at a relatively low pressure of 23 MPa and a temperature of 73 °C. At those conditions, a concentration of 172 g oleic acid/kg extract, 46 g linoleic acid/kg extract, and 5 g α-linolenic acid/kg extract would be achieved. For the benefit of extraction and our proposal, the optimal extraction conditions for the three fatty acids analyzed were the same, because the three target compounds belong to the same chemical family (fatty acids with 18 carbons) [30], with similar chemical structure, molecular weight, volatility [18], and diffusion coefficients [34].

As a final remark, and for the future feasibility of this work at an industrial scale, authors would like to mention that when the yield or the selectivity of the supercritical extraction is low, or the operating pressure or CO_2_ consumption is to be reduced, a cosolvent (entrainer or modifier) may be added to overcome the poor solvent nature of scCO_2_ [35,36,37]. However, considering the economic and practical difficulties involved in choosing a cosolvent at supercritical conditions, theoretical evaluations must be implemented. In this regard, the Hansen solubility theory (HST) has arisen as a suitable tool to reduce the number of experiments for the selection of a proper cosolvent for scCO_2_ [37]. The Hansen approach can be used to predict the best cosolvent for scCO_2_ in the solubilization of bioactive compounds from natural matrices within a specific interval of operating conditions [35,36,37]. In this regard, and in order to increase the lipid fraction rich in fatty acids such as linoleic and α-linolenic acids, some studies have made calculations with several organic solvents used in food and pharmaceutical industries [35,36]. In their previous reports, Professor Lourdes Calvo’s Research Group in Madrid [35,36] reached a selective extraction of oleic, linoleic, and α-linolenic acids by using the Hansen approach with a supercritical homogeneous mixture with a cosolvent volume fraction of 5% (0.05 m^3^/m^3^). The authors correctly predicted the order of the cosolvent ability in a wide range of operating conditions suitable for supercritical extraction, finding that the cosolvent order depended on the fatty acid, but in general, ethanol was the best cosolvent to solubilize and therefore to selectively extract oleic, linoleic and α-linolenic acids with scCO_2_. The above, along with the valorization strategy proposed in this study, could represent a chance to improve the commercial opportunity of the process. Finally, on a possible industrial scale, CO_2_ is recirculated to the process, which, as it is well known [28], undoubtedly lowers the operating costs of the high-pressure process.

## 3. Materials and Methods

### 3.1. Reagents

Boron trifluoride (BF_3_, 1.3M in methanol, Sigma-Aldrich, Pasto, Colombia), hexane (99%, Sigma-Aldrich, Pasto, Colombia), methanol (HPLC, ≥99.9%, Sigma-Aldrich, Pasto, Colombia), sodium hydroxide (NaOH, ≥98%, pellets anhydrous, Sigma-Aldrich, Pasto, Colombia), sodium sulfate anhydrous (Na_2_SO_4_, Sigma-Aldrich, Pasto, Colombia), undecanoic acid (98%, Sigma-Aldrich, Pasto, Colombia), CO_2_ (99.9% purity, CRYOGAS, Pasto, Colombia), helium (≥99.99%, CRYOGAS, Pasto, Colombia), and nitrogen (≥99.99%, CRYOGAS, Pasto, Colombia) were used for this study. All materials were used as received.

### 3.2. Biomass

Mango (*M. indica* L.; *var.* Tommy Atkins) seed kernels were obtained as a by-product from INPADENA, a company devoted to the production of fruit pulp in the city of Pasto (Nariño, Colombia). Fresh seed kernels were provided with an approximate water mass fraction of 44% (0.44kg water/kg seed kernel). The moisture was measured in an oven (Digiheat, J.P. Selecta, S.A., Pasto, Colombia) at 103 °C until the weight did not vary more than 0.1%.

Considering that the initial solid conditions of the raw material to be submitted to supercritical fluid extraction are especially important to maximize the extraction yield and to improve the quality of the extract [25], the raw material was dried and milled before being subjected to the extraction process. For drying, a CST-800 tray dryer (FIQ S.A.S., Pasto, Colombia) was used at 50 °C for 8 h until a humidity of 6.8% in mass fraction was reached, which corresponded to the equilibrium moisture of the raw material with the air in Pasto. Afterward, seed kernels were milled with a hammer mill (TRAPP, Vieira, Jaraguá do Sul, Brazil). The average particle size of the ground kernels was 0.6 mm, which was measured using a sieve (PS-35 series 1182, Pinzuar, Pasto, Colombia) with the ASTM E11 sieve series, mesh 0.10–0.80 mm.

### 3.3. Supercritical Fluid Extraction

#### 3.3.1. Experimental Installation

scCO_2_ extractions from mango seed kernels were carried out in an SFE 500 system, which is an automated supercritical fluid extraction apparatus from the Waters^®^ line of pilot-scale systems (Waters Acquires Thar Instruments, Milford, CT, USA). The experimental installation consists of a CO_2_ pump with an operating pressure capacity of up to 60 MPa, which is fed with CO_2_ at about 5.7 MPa from a cylinder with a dip tube. A cooling heat exchanger is used to cool and liquefy CO_2_ before it enters the pump, to avoid CO_2_ cavitation during pumping, and to guarantee maximum efficiency. An electrical heating heat exchanger is located upstream from a 0.5 L stainless steel vessel, to ensure that the CO_2_ is heated up before entering the extractor, which is equipped with a high-pressure valve to isolate the vessel from high pressures. It also has a cap with a spring-loaded seal that enhances safety and lends to automation for efficient loading and unloading. A mass flow meter, located on the inlet of the CO_2_ pump, measures liquified CO_2_ mass output. The system controls pressure by using a motor-driven automated back pressure regulator (ABPR), which is temperature-controlled to compensate for cooling during depressurization due to the Joule-Thomson effect. A built-in pressure sensor provides closed-loop feedback for control and pressure alarm monitoring through the software Process Suite V 5.9. Meanwhile, a temperature control monitors and controls up to six temperature zones independently. Moreover, the SFE 500 system is equipped with a high-pressure collection vessel.

**Figure 4:**
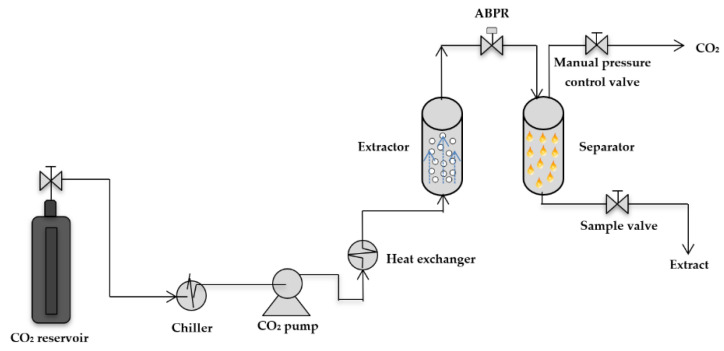
Schematic representation of the equipment used for supercritical fluid extraction.

CO_2_ is a non-toxic gas; however, it will displace the air in the room and can lead to suffocation if not properly ventilated. Therefore, the system also provides for venting to a fume hood through a 1/4’’ compression fitting and line. A schematic representation of the equipment is shown in Figure 4 and complete details of the equipment have been described in a previous report [38].

#### 3.3.2. Operation Procedure

The extractor was filled with 0.2 kg of dried and milled seed kernels, forming a fixed bed. The extractor was then closed, and the fluid was pumped in to achieve working conditions. After this, the ABPR was opened to provide a continuous flow of 30 g CO_2_/min through the bed for 150 min (4.5 kg of CO_2_). Previous experiments performed at the central point of the experimental design (30 MPa and 63 °C) revealed that a solvent ratio of 22.5 kg CO_2_/kg biomass was enough to deplete the plant material by up to 80% of its total oily extract content. After the ABPR, the fluid was depressurized; the solvent power of the CO_2_ dropped, the extract was collected in pre-weighed amber vials and kept at 4 °C until further analysis.

All the experiments were carried out at least two times and the experimental error was deduced from selected tests that were repeated six times. The oil extraction yield was expressed in g extract/kg biomass.

#### 3.3.3. Optimization

A Box–Wilson RCCD, containing an embedded factorial design 2^2^, with center points augmented with a group of star points allowed the estimation of curvature (RSM) to obtain the highest oil and EFAs yield. The independent factors studied to optimize the extraction yield (Y), along with their operating ranges, were pressure (X_1_; from 25–35 MPa) and temperature (X_2_; 55–70 °C). Levels of each factor were established from preliminary tests and coded as Table 3 shows.

Four (4) points from the factorial design, four (4) star points (α = 1.414), and four (4) central points (30 MPa and 63 °C in Table 1) were used for a total of twelve (12) experimental runs. All experiments were performed in randomized order to minimize the effect of unexplained variability in the observed responses due to the extraneous factors. A second-order polynomial regression equation was used to predict the response variable (Y) according to Equation (2) as follows:

(2) $$Y=\beta_{0}+\beta_{1}X_{1}+\beta_{2}X_{2}+\beta_{12}X_{1}X_{2}+\beta_{11\;}X_{1}^{2}+\beta_{22}X_{2}^{2}+\varepsilon$$

where Y is the response variable (extraction yield, g/kg); X_1_ and X_2_ are the independent factors pressure and temperature, respectively; β_0_ is the intercept; β_1_ and β_2_ are linear effect coefficients; β_11_ and β_22_ are quadratic effect coefficients; β_12_ is the coefficient for the interaction of factors; and ε is the random error. STATGRAPHICS Centurion XV.II (Statpoint Technologies, Inc., The Plains, VA, USA) was used for the optimization, analysis of experimental data, as well as for the creation of the design matrix. Data were submitted to ANOVA and the statistical significance of the factors was set at a significance level of 5% (*p* ≤ 0.05).

### 3.4. Conventional Solvent Extraction

To obtain the total extract yield and for comparison purposes, a Soxhlet extraction was performed using 0.01kg of dried and milled seed kernels. The sample was transferred into a cellulose thimble and inserted into a Soxhlet assembly fitted with a 250 mL flask. Then, 100 mL of hexane was added and the whole assembly was heated for 8h using a heating jacket. Afterwards, the extract was concentrated using a rotary evaporator Re 121 (BUCHI, Flawil, Switzerland). Solvent traces were removed in an oven at 103 °C for 2 h; and finally, the extract was stored in a refrigerator at 4 °C until further analysis.

### 3.5. Fatty Acids Analysis

To analyze the fatty acid profile of lipidic fractions from both supercritical and conventional extraction, fatty acids were first, transesterified and then related to its respective fatty acid methyl ester (FAME, Restek, Bellefonte, PA, USA). The derivatization was conducted following the method described by the AOAC Official Method 969.33 [39]. For this, approximately 50 mg of mango seed kernel oil was placed into a screw cap glass tube. As much as 10 mg of undecanoic acid (internal standard) and 1 mL of NaOH in methanol (0.5 N) were added into the tube and then nitrogen was blown for 15 s. The tube was covered tightly, vortexed, heated in a water bath for 5 min at 85 °C, and then cooled. Afterward, 1 mL of BF_3_ solution was added, vortexed and then nitrogen was blown to the tube. The tube was covered tightly, heated for 15 min at 85 °C, and then cooled. In this stage, fatty acids were converted to FAMEs. The extracting solvent, 3 mL of hexane, was added and then the mix was vortexed. Then 3 mL of saturated NaCl solution was added, and the solution was vortexed. Finally, the upper layer was transferred through a small amount of anhydrous Na_2_SO_4_ (placed on the top of a filter liner) into a test tube with a Pasteur pipette.

Subsequently, the samples were analyzed by gas chromatography (GC) with a Shimadzu GC-17A Version 3 (Shimadzu Corporation, Kyoto, Japan) equipped with a Flame Ionization Detector (FID) and a DB-WAX capillary column (30 m × 0.25 mm i.d. × 0.25 µm film thickness, J&W Scientific, Inc., Folsom, CA, USA) 5% phenyl-methylpolysiloxane. The separation was carried out with helium (1 mL/min) as a carrier gas. The column temperature was set at 40 °C for 5 min and then heated to 250 °C at 5 °C/min. A split injector (1:10) at 250 °C was used. The FID was also heated up to 280 °C. The injection volume was 1 μL.

The chromatographic data were obtained and processed with the Shimadzu Class Vp 4.3 Software and the compounds were quantified by relating them to the area of the internal standard.

## 4. Conclusions and Perspectives

This work explored the possibility of taking advantage of mango seed kernels as a by-product of the Colombian agroindustrial sector, to obtain a lipid fraction rich in both linoleic and α-linolenic acids, along with oleic acid. This could be a potentially valuable product for use in the cosmetic, pharmaceutical, and perhaps food industry since it would be obtained without the intervention of organic solvents. These supercritical extracts, which can be considered premium grade [33], could be used in the food industry as antimicrobial extracts and thus increase the shelf life of foods; but there is also a clear potential use as vegetable oil or additive in the confectionery industry for chocolates and bakery products [40]. Not to mention that a mixture of supercritical mango seed kernel extracts and palm stearin could also be used in countries with warm climates by chocolate manufacturers for its resistance to high temperatures [33]. The aforementioned gives a bioactive potential to mango seed kernels, developing a green valorization strategy and posing a great challenge and a unique opportunity for the mango processing industry in Colombia to deliver a value-added product to the market with health-promoting properties, based on a circular economy model.

The proposal made in this study required drying and milling of the raw material, expensive operations that associated with the high installation costs of the supercritical plant could jeopardize the economic viability of the process. Nonetheless, a quality oil with a clear potential use in nutraceutical formulations, cosmetics, or even high value-added drugs was obtained. Such an oil would not require downstream processing, and a revalorized biomass would be used, which, along with CO_2_ recycling on an industrial scale, would represent a strong contribution to the feasibility of the current proposal.

## Figures and Tables

**Table 1:** Design conditions and supercritical extraction yields from mango seed kernels.

Pressure (MPa)	Temperature (°C)	Extraction Yield (g Extract/kg Biomass)
**25 (−1)**	55 (−1)	50 ± 3 ^b^
**25 (−1)**	70 (+1)	35 ± 3 ^a^
**35 (+1)**	55 (−1)	69 ± 1 ^cd^
**35 (+1)**	70 (+1)	74 ± 2 ^cd^
**23 (−1.41)**	63 (0)	24 ± 1 ^a^
**37 (+1.41)**	63 (0)	83 ± 2 ^de^
**30 (0)**	52 (−1.41)	63 ± 2 ^bc^
**30 (0)**	73 (+1.41)	65 ± 1 ^bc^
**30 (0)**	63 (0)	73 ± 3 ^cd^

Different letters (a, b, c, d, and e) in the same column represent statistically significant differences at 5% significance.

**Table 2:** Fatty acid profile of mango seed kernel extracts obtained by supercritical and conventional solvent extraction.

Pressure (MPa)	Temperature (°C)	g Fatty Acid/kg Extract					SFA/UFA
		C_16:0_	C_18:0_	C_18:1_	C_18:2_	C_18:3_	
25	55	32 ± 1 ^abc^	61 ± 2 ^ab^	93 ± 1 ^ab^	24 ± 1 ^ab^	3 ± 0 ^bcd^	1.29 ^a^
25	70	38 ± 2 ^cd^	63 ± 1 ^ab^	105 ± 1 ^abc^	31 ± 1 ^ab^	4 ± 0 ^de^	1.41 ^a^
35	55	28 ± 1 ^abc^	45 ± 1 ^a^	139 ± 1 ^bc^	25 ± 0 ^ab^	2 ± 0 ^abc^	2.45 ^a^
35	70	23 ± 1 ^ab^	54 ± 1 ^ab^	69 ± 0 ^a^	16 ± 1 ^a^	1 ± 0 ^a^	1.12 ^a^
23	63	50 ± 1 ^d^	71 ± 1 ^ab^	155 ± 1 ^c^	37 ± 2 ^b^	4 ± 0 ^e^	1.62 ^a^
37	63	23 ± 1 ^ab^	55 ± 2 ^ab^	69 ± 1 ^a^	16 ± 1 ^ab^	2 ± 0 ^ab^	1.09 ^a^
30	52	35 ± 1 ^bc^	84 ± 1 ^b^	111 ± 1 ^abc^	24 ± 1 ^ab^	2 ± 0 ^ab^	1.14 ^a^
30	73	41 ± 1 ^cd^	64 ± 1 ^ab^	111 ± 2 ^abc^	33 ± 1 ^ab^	3 ± 0 ^cde^	1.41 ^a^
30	63	30 ± 1 ^abc^	62 ± 1 ^ab^	93 ± 1 ^abc^	22 ± 1 ^ab^	2 ± 0 ^abc^	1.25 ^a^
Soxhlet extraction		21 ± 1 ^a^	47 ± 1 ^a^	61 ± 2 ^a^	17 ± 1 ^ab^	2 ± 0 ^ab^	1.15 ^a^

Palmitic (C_16:0_), stearic (C_18:0_), oleic (C_18:1_), linoleic (C_18:2_) and α-linolenic (C_18:3_) acid. Ratio saturated/unsaturated fatty acids. Different letters (a, b, c, d, and e) in the same column represent statistically significant differences at 5% significance.

**Table 3:** Factors and levels of the rotatable central composite design.

Factor	Level		
	Low (−1)	Central (0)	High (+1)
**Pressure (MPa, X_1_)**	25	30	35
**Temperature (°C, X_2_)**	55	63	70

## Data Availability

The data used to support the findings of this study are available from the corresponding author upon request.

## Sample Availability

Limited amounts of the compounds are available at the Emerging Technologies in Agroindustry Research Group from the Universidad de Nariño.

## References

[1] Imbert E. (2017). Food waste valorization options: Opportunities from the bioeconomy. Open Agric..

[2] Sánchez-Camargo A., Gutiérrez L.F., Vargas S.M., Martinez-Correa H.A., Parada-Alfonso F., Narváez-Cuenca C.E. (2019). Valorisation of mango peel: Proximate composition, supercritical fluid extraction of carotenoids, and application as an antioxidant additive for an edible oil. J. Supercrit. Fluids.

[3] Otles S., Kartal C. (2018). Food waste valorization. Sustainable Food Systems from Agriculture to Industry.

[4] Wang H.W., Liu Y.Q., Wei S.L., Yan Z.J., Lu K. (2010). Comparison of microwave-assisted and conventional hydrodistillation in the extraction of essential oils from mango (*Mangifera indica* L.) flowers. Molecules.

[5] Severi J.A., Lima Z.P., Kushima H., Monteiro Souza Brito A.R., Campaner dos Santos L., Vilegas W., Hiruma-Lima C.A. (2009). Polyphenols with antiulcerogenic action from aqueous decoction of mango leaves (*Mangifera indica* L.). Molecules.

[6] FAO (2020). Medium-Term Outlook: Prospects for Global Production and Trade in Bananas and Tropical Fruits 2019 to 2028.

[7] Sánchez-Camargo A., Ballesteros-Vivas D., Buelvas-Puello L.M., Martinez-Correa H.A., Parada-Alfonso F., Cifuentes A., Ferreira S.R.S., Gutiérrez L.F. (2021). Microwave-assisted extraction of phenolic compounds with antioxidant and anti-proliferative activities from supercritical CO_2_ pre-extracted mango peel as valorization strategy. LWT.

[8] Ballesteros-Vivas D., Álvarez-Rivera G., Morantes S.J., Sánchez-Camargo A., Ibáñez E., Parada-Alfonso F., Cifuentes A. (2019). An integrated approach for the valorization of mango seed kernel: Efficient extraction solvent selection, phytochemical profiling and antiproliferative activity assessment. Food Res. Int..

[9] Minagricultura (2019). Cadena del Mango: Indicadores e Instrumentos.

[10] Ballesteros-Vivas D., Alvarez-Rivera G., García Ocampo A.F., Morantes S.J., Sánchez-Camargo A., Cifuentes A., Parada-Alfonso F., Ibánez E. (2019). Supercritical antisolvent fractionation as a tool for enhancing antiproliferative activity of mango seed kernel extracts against colon cancer cells. J. Supercrit. Fluids.

[11] Gasiński A., Kawa-Rygielska J., Szumny A., Czubaszek A., Gąsior J., Pietrzak W. (2020). Volatile compounds content, physicochemical parameters, and antioxidant activity of beers with addition of mango fruit (*Mangifera indica*). Molecules.

[12] Garrido-Suárez B.B., Garrido G., Delgado R., Bosch F., Rabí M.d.C. (2010). A *Mangifera indica* L. extract could be used to treat neuropathic pain and implication of mangiferin. Molecules.

[13] Khaw K.-Y., Parat M.-O., Shaw P.N., Falconer J.R. (2017). Solvent supercritical fluid technologies to extract bioactive compounds from natural sources: A review. Molecules.

[14] Uwineza P.A., Waśkiewicz A. (2020). Recent advances in supercritical fluid extraction of natural bioactive compounds from natural plant materials. Molecules.

[15] Reverchon E., De Marco I. (2006). Supercritical fluid extraction and fractionation of natural matter. J. Supercrit. Fluids.

[16] Tyśkiewicz K., Konkol M., Rój E. (2018). The application of supercritical fluid extraction in phenolic compounds isolation from natural plant materials. Molecules.

[17] Durante M., Ferramosca A., Treppiccione L., Di Giacomo M., Zara V., Montefusco A., Piro G., Mita G., Bergamo P., Lenucci M.S. (2020). Application of response surface methodology (RSM) for the optimization of supercritical CO_2_ extraction of oil from patè olive cake: Yield, content of bioactive molecules and biological effects in vivo. Food Chem..

[18] Rustan A.C., Drevon C.A. (2005). Fatty acids: Structures and properties. Encyclopedia of Life Sciences.

[19] Serafim V., Tiugan D.-A., Andreescu N., Mihailescu A., Paul C., Velea I., Puiu M., Niculescu M. (2019). Development and validation of a LC–MS/MS-based assay for quantification of free and total omega 3 and 6 fatty acids from human plasma. Molecules.

[20] Kaur N., Chugh V., Gupta A.K. (2014). Essential fatty acids as functional components of foods—A review. J. Food Sci. Technol..

[21] BCC Research (2019). Oleochemical Fatty Acids: Global Markets to 2023.

[22] Awolu O.O., Manohar B. (2019). Quantitative and qualitative characterization of mango kernel seed oil extracted using supercritical CO_2_ and solvent extraction techniques. Heliyon.

[23] Jahurul M.H.A., Zaidul I.S.M., Norulaini N.N.A., Sahena F., Jaffri J.M., Omar A.K.M. (2014). Supercritical carbon dioxide extraction and studies of mango seed kernel for cocoa butter analogy fats. CyTA J. Food.

[24] Jahurul M.H.A., Zaidul I.S.M., Norulaini N., Ferdosh S., Rahman M.M., Mohd A.K. (2015). Optimization of supercritical carbon dioxide extraction parameters of cocoa butter analogy fat from mango seed kernel oil using response surface methodology. J. Food Sci. Technol..

[25] Tirado D.F., de la Fuente E., Calvo L. (2019). A selective extraction of hydroxytyrosol rich olive oil from alperujo. J. Food Eng..

[26] Grodowska K., Parczewski A. (2010). Organic solvents in the pharmaceutical industry. Acta Pol. Pharm..

[27] Viguera M., Marti A., Masca F., Prieto C., Calvo L. (2016). The process parameters and solid conditions that affect the supercritical CO_2_ extraction of the lipids produced by microalgae. J. Supercrit. Fluids.

[28] Brunner G. (1994). Gas Extraction.

[29] Del Valle J.M., De La Fuente J.C. (2006). Supercritical CO_2_ extraction of oilseeds: Review of kinetic and equilibrium models. Crit. Rev. Food Sci. Nutr..

[30] Gurr M.I., Harwood J.L. (1991). Fatty acid structure and metabolism. Lipid Biochemistry.

[31] Simopoulos A.P. (1999). Essential fatty acids in health and chronic disease. Am. J. Clin. Nutr..

[32] Lieb V.M., Schuster L.K., Kronmüller A., Schmarr H.-G., Carle R., Steingass C.B. (2019). Fatty acids, triacylglycerols, and thermal behaviour of various mango (*Mangifera indica* L.) kernel fats. Food Res. Int..

[33] Torres-León C., Rojas R., Contreras-Esquivel J.C., Serna-Cock L., Belmares-Cerda R.E., Aguilar C.N. (2016). Mango seed: Functional and nutritional properties. Trends Food Sci. Technol..

[34] López-Padilla A., Ruiz-Rodriguez A., Reglero G., Fornari T. (2016). Study of the diffusion coefficient of solute-type extracts in supercritical carbon dioxide: Volatile oils, fatty acids and fixed oils. J. Supercrit. Fluids.

[35] Tirado D.F., Rousset A., Calvo L. (2019). The selective supercritical extraction of high-value fatty acids from *Tetraselmis suecica* using the Hansen solubility theory. Chem. Eng. Trans..

[36] Tirado D.F., Tenorio M.J., Cabañas A., Calvo L. (2018). Prediction of the best cosolvents to solubilise fatty acids in supercritical CO_2_ using the Hansen solubility theory. Chem. Eng. Sci..

[37] Tirado D.F., Calvo L. (2019). The Hansen theory to choose the best cosolvent for supercritical CO_2_ extraction of β-carotene from *Dunaliella salina*. J. Supercrit. Fluids.

[38] Cerón L.J., Hurtado A.M., Ayala A.A. (2016). Efecto de la presión y la temperatura de extracción con CO_2_ supercrítico sobre el rendimiento y composición de aceite de semillas de guayaba (*Psidium guajava*). Inf. Tecnológica.

[39] (1995). AOAC Fatty acid in oils and fats preparation of methyl ester boron trifluoride method. Official Methods of Analysis of AOAC International.

[40] Torres-León C., Ascacio-Valdés J.A., Chávez-González M.L., Serna-Cock L., Ramirez-Guzman N., Cintra A., López-Badillo C., Rojas R., Belmares-Cerda R., Aguilar C.N., Flores-Gallegos A.C., Rodriguez-Jasso R.M., Aguilar C.N. (2020). Valorization of Ataulfo mango seed byproduct based on its nutritional and functional properties. Bioprocessing of Agri-Food Residues for Production of Bioproducts.

